# A Study of the Chemiluminescence of the Pb + O_3_ Reactions*

**DOI:** 10.6028/jres.080A.018

**Published:** 1976-04-01

**Authors:** M. J. Kurylo, W. Braun, S. Abramowitz, M. Krauss

**Affiliations:** Institute for Materials Research, National Bureau of Standards, Washington, D.C. 20234

**Keywords:** Chemiluminescence, electronic states, gas kinetics, laser enhanced reactions, O_3_: PbO

## Abstract

The chemiluminescent reaction of Pb + O_3_ has been studied using both “cold” and vibrationally excited O_3_. Emission from new states *a* and *b* has been observed in addition to the A and B states. The reaction of vibrationally excited O_3_ with Pb to yield PbO(A) appears to be faster than that using “cold” O_3_.

## 1. Introduction

The results of our recent studies on laser enhanced reactions have shown that vibrational energy in a reactant molecule can reappear as excitation in a product species. For example, emissions from both ${\text{NO}}_{2}^{*}$ and ${\text{SO}}_{2}^{*}$ produced in the reactions of NO and SO with O_3_ were observed to shift to shorter wavelength when the O_3_ was vibrationally excited using a CO_2_ laser [1, 2].^1^ Because of the spectroscopic complexities of the triatomic molecules, NO_2_ and SO_2_, our intention in the present study was to investigate a chemiluminescent reaction producing a diatomic product which would be, in principle, easier to characterize spectro-scopically. The metal atom-oxidant systems represent a class of such reactions. A significant number of these have been investigated to date because of their potential as chemical lasers. We report herein some observations on the reaction

$$\text{Pb}+O_{3}\to \text{PbO}+O_{2}.$$

While the information obtained from studying the infrared laser-enhanced reaction component is minimized by the overall reaction complexity, it does nevertheless provide some additional insight into this reaction system. This information coupled with new high pressure spectroscopic results complement the detailed low pressure investigation by Oldenborg, Dickson, and Zare (ODZ) [3].

## 2. Experimental Detail

**Figure 1:**
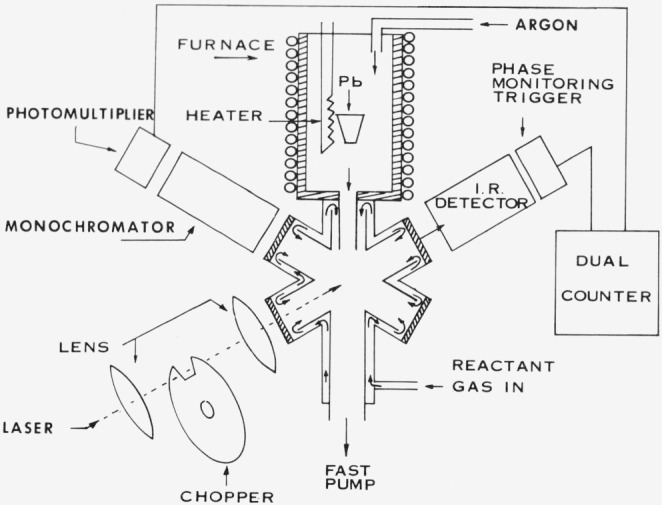
Schematic of the chemiluminescent flow reaction apparatus.

The furnace, reaction cell, fast flow pumping system, CO_2_ laser, and spectrometer are shown schematically in figure 1. Lead vapor, produced from a resistively heated crucible containing lead metal enters the glass reaction chamber in an Ar diluent stream. The effluent from a commercial O_3_ generator, consisting of 4 percent O_3_ in O_2_ is mixed with Ar and flows past the cell windows (to eliminate window deposits) into the cell. There it diffusively mixes with the lead-argon flow. The temperature of the crucible (as measured by a thermocouple probe) ranges from 900–1000 K while the temperature in the flame reaction zone varies from 500–600 K. The total pressure in the cell varies from 1 to 5 torr (1 torr = 133.3 Pa) with the flow through the furnace being anywhere from 20 to 50 percent of the total flow.

The 0.5 cm diameter beam from a CO_2_ laser tuned to the 9.6 *μ*m P(30) transition is square wave chopped and traverses the flame exciting *v*_3_, the asymmetric stretching mode of O_3_ (1043 cm^−1^). The chemiluminescence from the Pb + O_3_ reaction is monitored through a spectrometer-photomultiplier assembly. The photomultiplier output is fed through a series of pulse amplifiers and voltage discriminator into a dual counter, one channel of which records the “laser-on” signal and the other the “laser-off”. A printout from these counters is synchronized with the spectrometer wavelength-scan-drive thereby facilitating the recording of the modulation spectrum vs wavelength. The normal spectral (“laser-off”) emission is automatically obtained from the “laser-off” counter.

## 3. Results

**Figure 2:**
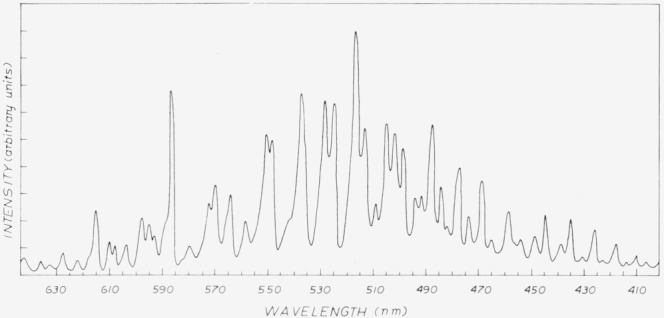
Chemiluminescence spectrum for the *Pb*(*v*) + *O*_3_ reaction.

In contrast to the spatially sharp diffusion flame observed for the Ba + O_3_ (or N_2_O) reaction, the Pb + O_3_ flame is quite diffuse (apparently reaction limited). It is brightest in the high temperature zone at the furnace nozzle, diminishes with decreasing temperature (increasing distance from the nozzle), and persists for some two to three feet into the pumping system. These observations suggest a reaction rate for Pb + O_3_ which is considerably slower than gas kinetic. The spectrum which we observe at several torr total pressure partly resembles the low pressure spectrum obtained by ODZ and reproduced by us in a similar quasi-beam apparatus. The short wavelength end of the more complete spectrum (fig. 2) can be identified from the low (submicron) pressure spectrum in that it is less diffuse. The long wavelength portion between 480 nm and 595 nm is almost entirely the new *a* state recently characterized by ODZ. At still longer wavelengths, our spectrum differs from that of ODZ in that we observe a series of strong lines which do not agree with *a* → X, A → X, or B → X, but rather appear to originate from a new state observed via six weak lines by ODZ and identified as *b* by them. This *b* state is seen more intensely in our high pressure spectrum. This series of lines in our spectrum can be fit to the expression:

$$v(v^{\prime },v^{\prime \prime })=16315+441.0v^{\prime }-\{717.7v^{\prime \prime }-3.53{v^{\prime \prime }}^{2}\}$$

where the lower state constants have been taken from Rosen’s compendium [4]. The spectral constants given here and in tables 1 and 2 for the *a*, A, and B state are not as well determined as previous values given in references [1] and [4].

A definite vibrational assignment for an electronic state, for which a rotational analysis has not been observed, requires the measurement of a vibrational isotope shift. Lacking data of this type one tries to assign band heads such that both (0, *v*″) and (*v*′, 0) levels are observed. The assignment hypothesized by ODZ on the basis of six observed transitions in a (*v*′, 0) transition has placed *T*_00_ of the *b* state about one vibrational quanta above *T*_00_ of the *a* state. In this study 20 band heads have been observed that can be assigned to *b* state. In addition to the (*v*′, 0) band heads, other bands have been observed with *v*″=l, 2, 3, 4, 5. The hypothesis regarding the separation of the *a* and B states is supported by arguments given later.

Although considerable intensity is observed for B — X transition, the zeroth level of this state lies very close to the thermodynamic threshold for the reaction

$$\text{Pb}(J=0)+O_{3}\to \text{PbO}(B)+O_{2}.$$

The dissociation energy $\left(D_{0}^{\circ }\right)$ of PbO is given by Rosen [4] as 30,920±500 cm^−1^. Using Δ*Hf*°(298 K) of O_3_ and O as 34.1 and 59.5 kcal/mol as recommended by Wagman et al. [5], one computes 22036 cm^−1^ for the exothermicity of the reaction.

$$\text{Pb}(J=0)+O_{3}\to \text{PbO}(X^{\prime }\Sigma^{+})+O_{2}$$

This to be compared to 22174 cm^−1^ of the *v*_(0,0)_ level of the B state. Oldenborg and Zare have suggested that production of this state results from reaction of Pb (*J*=1) present in their experiments due to an electric discharge in their furnace. The strong B → X emission observed in our high pressure experiment was found to vary markedly with the oven temperature. The observed B → X emission increase over that emission from either *a* or *b* → X with increasing temperature obeyed an Arrhenius-type formulation with an activation energy of some 10 kcal (i.e. nearly equal to the energy difference between *J* = 0 and *J* = 1 multiplet components of Pb). This observation suggests that Pb (*J* = 1) is produced thermally in our furnace as opposed to its possible production via secondary processes, e. g.

PbO*+Pb(J=0)→PbO+Pb(J=1).

If PbO(B) is indeed produced through reaction of Pb (*J* = 1) with ozone, the rate of this reaction must be extremely fast to compete with the deactivation process

$$\text{Pb}(J=1)+O_{2}\to \text{Pb}(J=0)+O_{2}$$

since the deactivation proceeds with a high rate constant and the O_2_ pressure is twenty times that of O_3_ under our usual experimental conditions.

**Figure 3:**
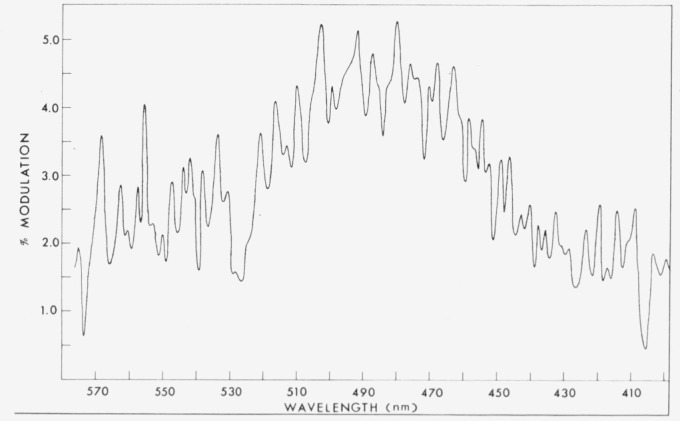
Smoothed modulation spectrum (the component of the emission which varies with laser excitation of *O*_3_) of the *Pb*(*v*) + *O*_3_ reaction.

Part of the modulation spectrum is reproduced in figure 3. This spectrum, which has been smoothed, represents only that component of the chemiluminescent emission which varies with the laser excitation of O_3_ (i.e. only that component which either increases or decreases due to a change in reaction rate with reactant vibrational excitation). Thus, if the emission originated only from a single electronic state, the modulation spectrum would appear as a quasi-continuum with possibly some small structure due to sharp changes in population of one vibrational line relative to an adjacent one. The large amount of structure in figure 3 indicates the presence of emission from at least two electronic states. One might speculate that at the longer wavelength, where the normal spectrum consists mainly of *a* and *b* emission lines, this modulation structure could reveal an increase or decrease of *b* emission lines relative to *a* emission lines (or vice versa). However, the peaks (or valleys) in the modulation spectrum do not agree well with either *a* or *b* state emission lines. More surprisingly, they agree much better with A → X transitions. We therefore tentatively identify the modulation spectrum as a quasi continuum due to *a* → X and *b* → X emissions at long wavelengths (possibly some B → X short wavelength) with structure due to A → X emission lines superimposed throughout the entire wavelength range. This suggests that vibrational excitation in O_3_ increases the production of the *a, b*, and possibly the B state of the PbO all to some small degree, while increasing A state production most significantly. We must emphasize, however, that the assignment of A state responsibility for structure in the modulation spectrum is tentative since the modulation data are difficult to obtain, require long integration times, and should be obtained in the low (submicron) pressure region where only the *a* and B states have been observed. Nevertheless, we can speculate on the production of PbO (A) in the laser excited reaction. It is possible that as a result of laser excitation of O3 forming O_3_† the reaction with Pb (*J* = 1) now produces PbO (B) in higher vibrational levels. These can then be collisionally transferred to the A state which is seen in emission. A second equally likely possibility is that the vibrational energy in O_3_ increases the rate of A state production directly at the expense of one or more of the other reaction channels.

From the numerical value of the percent modulation in the laser experiments (i.e. the continuum region assigned to *a* and *b* state enhancement) we can calculate a rate constant enhancement of approximately a factor of two. This is obtained by comparing the percent modulation of both the Pb+O_3_† and NO+O_3_† reactions in this apparatus under identical experimental conditions.

We can obtain a very rough estimate of the quantum yield of *a* and *b* state production in the following manner. First we assume the reaction of O_3_ with Pb (*J* = 1) funnels entirely into PbO (B), (i.e. quantum yield of unity). From figure 2 we see that the *a*+*b* emission intensity is comparable to the intensity of PbO (B). However, at the oven temperature of 1000 K only 1 part of 10^5^ of the Pb atoms are in *J* = 1. This would imply a quantum yield of *a* + *b* due to Pb (*J* = 0) of about 10^−5^ assuming Pb (*J* = 0) and Pb (*J* = 1) reacted with O_3_ with the same rate constant. The observation of the decay of chemiluminescence over several feet in our flow tube points to a slow rate of reaction for Pb (*J* = 0) while the fact that the reaction of Pb (*J* = 1) must compete favorably with its deactivation channels speaks for a very fast (collision frequency) rate of reaction for Pb (*J* = 1). Assuming a reaction efficiency of 10^−2^ for the Pb (*J* = 0) reaction in the spectrometer observation zone increases the quantum yield for *a* + *b* state production by a factor of 100 to 10^−3^. We compare this to the ODZ upper limit of 10^−2^ obtained under the assumption of a fast reaction rate for Pb (*J* = 0). A slower reaction rate would unfortunately raise their quantum yield (in greater disagreement with our 10^−3^ estimate). However, our observation of a large luminescent region is not necessarily contradictory to ODZ’s estimate of a fast reaction rate because of the different temperature regions of the two studies. In the quasi-beam configuration, Pb exits the furnace at very high temperature (> 1000 K) and collides with O_3_ with much higher kinetic energy than in our high pressure experiments (kinetic energy of 600 K at the nozzle tip and decreasing rapidly to 400 K downstream of the mixing zone).

## 4. Analysis and Conclusions

Oldenborg et al. identify the two (0^−^, 1) components of the ^3^Σ^+^ as *b* and *a*, respectively. This energy ordering is consistent with the present observations including an analysis of the relative intensities of the *a* and *b* transitions. A theoretical analysis can also be made of the spin-spin splitting constant of the ^3^Σ state which shows that the 0^−^ component must be at a higher energy than the 1 component. The spin-spin splitting for a molecule containing a heavy atom is dominated by the second-order spin-orbit coupling to nearby electronic states. Spin-orbit coupling will occur for ΔΩ = 0 and couple the 0^−^ component to ^3^*π* (0^−^), ^1^Σ^−^(0^−^) and the 1 component to ^3^*π*(1), ^3^Σ^−^(1), ^1^*π*(1). Note that there is no first-order spin-orbit coupling between the ^3^Σ^+^(1) and ^3^Δ(1) states. Using a hamiltonian of the form

$$\sum_{i}l_{i}\cdot s_{i},$$

the spin-orbit interaction is reduced [6] to the one-electron spin-orbit integrals given in table 3.

Interaction with the ^3^*π* state shifts both components equally and can be ignored. The ^1^*π*(1) interaction is small both because the *σ* and *π* orbitals are primarily localized on the oxygen atom, and there is a large energy separation between the ^1^*π* and ^3^Σ^−^ states. The spin-spin splitting constant is dominated by the difference between the ^3^Σ^−^(1) and ^1^Σ^−^(0^−^) interactions. From table 3 we see that the ^3^Σ^−^ interaction is necessarily the largest term. This demonstrates that the ^3^Σ^−^(l) component is lower in enrgy than ^3^Σ^−^(0).

Quantitative estimates of the spin-spin splitting cannot be made from molecular spin-orbit parameters since little is known for the PbO excited states. Using the atomic spin-orbit parameter [6], assuming atomic populations by comparing calculations on PbO [7] and CO [8], and excitation energies for the perturbing states [9], a spin-spin splitting of about 1000 cm^−1^ is obtained. This qualitatively confirms the analysis of the emission spectrum and the assignment of the *a* and *b* states. Assuming that the molecular integrals will scale with the atomic spin-orbit parameters, the splitting in the case of SnO would be an order of magnitude less than the value for PbO while GeO is about two orders of magnitude less.

There are two mechanisms which could give intensity to the dipole forbidden *b → X* transition, spinrotation and electronic rotational coupling. For large spin-spin splitting the spin-rotation coupling between the *a* and *b* states is negligible even for *J* as large as 100 (one expects the most probable value of *J* to be 24 for *T* = 500 K).

The electronic-rotation coupling matrix elements between a ^3^Σ^+^ and ^3^*π* state are given by Kovacs [11]. The coupling is proportional to 4*B*^2^*J* (*J* + 1)/Δ*E* for the Hund’s case *a* representation where Δ*E* is the difference in the electronic energies and *B* is the rotational constant. Since Δ*E* ~ 5000 cm^−1^ and *T* ~ 500 K, this coupling is significant hut can only be ascertained quantitatively if the complete mixing between ^3^Σ^+^ and ^3^*π* states is considered. It is likely that the *b* state transition probability would increase with temperature.

Since all the observed transitions to the ground state ultimately derive their intensity from spin-orbit mixing to the higher singlet excited states, it is well to remember that the transitions between ^3^*π* and ^3^Σ^+^ states are electric-dipole allowed and should have significant intensity even though they are in the infrared (~5000 cm^−1^).

The present results point to a slow overall reaction of Pb (*J* = 0) with O_3_ as well as a small quantum yield for production of PbO excited electronic states. Since the reactant states can couple to only one final product state, adiabatically this must be PbO (X) and O_2_ (X). Excited PbO states are the result of a nonadiabatic transition. The enhanced reactivity of Pb (*J* = 1) into the excited B state reflects the additional channels that are now available but the population yield of the various excited states remains puzzling.

Our observations at high pressure are somewhat different from those of Oldenborg et al. In particular, we find more intensity in the *b* state than they did. Their observations were made before collisions took place. In the present case many collisions can occur before the emitted light is observed. Two general explanations can be advanced for the different observations. The effective rotational temperatures can be very different. Pressure effects can also be important both by collisionally deactivating other excited states into the *b* state or by collisionally inducing the radiative transition.

Finally, we note that the enhancement or even initial formation of the A state by laser excitation of the ozone is not understood. In order to further unravel these questions, additional experiments must be carried out in conjunction with a more extensive theoretical investigation of the Group IV oxides.

## Figures and Tables

**Table 1:** Observed band heads and assignments in cm^−1^

*v*(obs)	*v*(calc)	assignment
24143	24116	B (4.0)
23590	23640	B (3,0)
23392	23402	B (4,1)
23170	23157	B (2,0)
22957	22926	B (3,1)
22636	22665	B (1,0)
22457	22443	B (2,1)
22178	22166	B (0,0)
21954	21951	B (1,1)
21636	21660	*b* (12,0)
21474	21452	B (0,1)
21169	21211	*b* (11,0)
21079	21065	B (5,5)
20877	20870	*a* (11,0)
20721	20745	B (0,2)
20614	20616	B (4,5)
20442	20445	*a* (10,0)
20338	20314	*b* (9,0)
20149	20156	*a* (11,1)
20024	20014	*a* (9,0)
19904	19867	*b* (8,0)
19743	19731	*a* (10,1)
19585	19578	*a* (8,0)
19467	19449	*a* (11,2)
19128	19138	*a* (7,0)
19015	19023	*a* (10,2)
18688	18692	*a* (6,0)
18515	18530	*b* (5,0) Sh.
18305	18323	*a* (10,3)
18238	18241	*a* (5,0)
17986	17978	*a* (6,1)
17790	17785	*a* (4,0)
17615	17630	*a* (10,4)
17535	17527	*a* (5,1)
17319	17325	*a* (3,0)
17212	17199	*b* (2,0)
17108	17052	*b* (8,4)
17033	17108	*b* (5,2)
16915	16853	*b* (6,3)
16858	16859	*a* (2,0)
16773	16757	*b* (1,0)
16614	16664	*b* (4,2)
16488	16485	*b* (2,1)
16434	16367	*b* (10,5)
16297	16315	*b* (0,0)
16215	16220	*b* (3,2)
16108	16160	*b* (6,4)
15773	15778	*b* (2,2)
15623	15601	*b* (0,1)
15672	15715	*b* (5,4)
15363	15335	*b* (1,2)

The calculated *v* have been generated from a least-squares treatment of the observed data using Rosen’s constants for the X state. The generating equations are:
$$\begin{array}{l} a.\;v_{\left(v^{\prime },v^{\prime \prime }\right)}=15912+478.4v^{\prime }-2.5{v^{\prime }}^{2}-\{717.7v^{\prime \prime }-3.53{v^{\prime \prime }}^{2}\}\\ b.\;v_{\left(v^{\prime },v^{\prime \prime }\right)}=16315+441.0v^{\prime }-\{717.7v^{\prime \prime }-3.53{v^{\prime \prime }}^{2}\}\\ c.\;v_{\left(v^{\prime },v^{\prime \prime }\right)}=22166+502.0v^{\prime }-3.8{v^{\prime }}^{2}-\{717.7v^{\prime \prime }-3.53{v^{\prime \prime }}^{2}\} \end{array}$$

**Table 2:** Observed band heads for the modulated spectrum (*A* → *X*) in cm^−1^

*v*(obs)	*v*(calc)	*v*′	*v*″
17592	17599.3	0	3
18319	18299.3	0	2
19015	19006.4	0	1
19708	19720.6	0	0
18005	18041.	1	3
18748	18741.	1	2
17765	17789.3	2	4
18470	18482.3	2	3
19216	19182.4	2	2
19885	19889.5	2	1
20644	20603.6	2	0
18925	18923.2	3	3
19611	19623.3	3	2
20350	20330.4	3	1
17972	17984.9	4	5
18642	18670.8	4	4
19384	19363.8	4	3
20064	20063.8	4	2
20773	20770.9	4	1
21463	21485.1	4	0
19826	19803.9	5	3
20517	20504.	5	2
21191	21211.1	5	1
18783	18864.8	6	5
19554	19550.7	6	4
20226	20243.7	6	3
20947	20943.8	6	2
21737	21650.9	6	1
20024	19990.1	7	4
18403	18392.5	8	7
19015	19064.3	8	6
20434	20429.1	8	4
18854	18831.	9	7
19497	19502.9	9	6
17940	17946.8	10	9
18573	18604.5	10	8
19271	19269.2	10	7
19964	19941.1	10	6
20584	20619.9	10	5
21350	21305.9	10	4

The calculated *v* have been generated from a least-squares treatment of the data using Rosen’s constants for the X state. The generating equation is:
$$v(v^{\prime },v^{\prime \prime })=19721+441.9v^{\prime }-0.20{v^{\prime }}^{2}-\{717.7v^{\prime \prime }-3.53{v^{\prime \prime }}^{2}\}$$

**Table 3:** Molecular spin-orbit interactions

Ω = *0*^−^
${\;}^{3}\Sigma^{+}{-}^{3}\pi -1/2\sqrt{2}<\pi ||\sigma >$
^3^Σ^+^− ^1^Σ^−^ − 1/2 < *π* || *π* > + 1/2 < π* || π* >
Ω = 1
${\;}^{3}\Sigma^{+}{-}^{3}\pi 1/2\sqrt{2}<\pi ||\sigma >$
${\;}^{3}\Sigma^{+}{-}^{1}\pi -1/2\sqrt{2}<\pi ||\sigma >$
^3^Σ^+^− ^3^Σ^−^ − 1/2 < *π* || *π* > − 1/2 < π* || π* >

## Footnotes

1 Figures in brackets indicate the literature references at the end of this paper.
